# *Planococcus* Species – An Imminent Resource to Explore Biosurfactant and Bioactive Metabolites for Industrial Applications

**DOI:** 10.3389/fbioe.2020.00996

**Published:** 2020-08-18

**Authors:** Samadhan Waghmode, Mangesh Suryavanshi, Deepansh Sharma, Surekha K. Satpute

**Affiliations:** ^1^Department of Microbiology, Elphinstone College, Mumbai, India; ^2^Yenepoya Research Centre, Yenepoya Deemed to be University, Mangalore, India; ^3^Amity Institute of Microbial Technology, Amity University, Rajasthan, India; ^4^Department of Microbiology, Savitribai Phule Pune University, Pune, India

**Keywords:** *Planococcus*, biosurfactant, genomic insight, glycolipid, marine, hydrocarbon degradation

## Abstract

The marine environment represents a well-off and diverse group of microbes, which offers an enormous natural bioactive compounds of commercial importance. These natural products have expanded rigorous awareness due to their widespread stability and functionality under harsh environmental conditions. The genus *Planococcus* is a halophilic bacterium known for the production of diverse secondary metabolites such as 2-acetamido-2-deoxy-α-d-glucopyranosyl-(1, 2)-β-d-fructofuranose exhibiting stabilizing effect and methyl glucosyl-3,4-dehydro-apo-8-lycopenoate displaying antioxidant activity. The genus *Planococcus* is reported generally for hydrocarbon degradation in comparison with biosurfactant/bioemulsifier secretion. Although *Planococcus* was proposed in 1894, it seized long stretch (till 1970) to get accommodated under the genus *Planococcus* authentically. Large-scale biosurfactant production from *Planococcus* was reported in 2014 with partial characterization. For the first time in 2019, we documented genomic and functional analysis of *Planococcus* sp. along with the physico-chemical properties of its biosurfactant. In 2020, again we screened biosurfactant for pharmacological applications. The present review discusses the comprehensive genomic insights and physical properties of *Planococcus*-derived biosurfactant. Moreover, we also highlight the prospects and challenges in biosurfactant production from *Planococcus* sp. Among ∼102 reports on biosurfactant produced by marine bacteria, 43 were of glycolipid and 59 were non-glycolipid type. Under other biosurfactant type, they were identified as lipopeptide (20) like surfactin (5), glycolipoprotein/lipoprotein (12), and other non-glycolipid (22). *Planococcus* sp. generally produces glycolipid-type biosurfactant (4) and exopolysaccharides (2). The single report documented in the literature is on biosurfactant production (glycolipid +non glycolipid) by diverse marine microbes (39) suggesting their novelty and diversity for biosurfactant secretion.

## Richness and Uniqueness of Marine Ecosystem, and Challenges Faced to Explore Its Microbial Wealth

Marine ecosystems represent the largest one among different aquatic ecosystems of our planet where high salinity makes it different in comparison with another aquatic reservoir (Wang and D’Odorico, 2013). Salty water resulted through several mechanisms like rain, rock and sediment erosion processes, exchange of gases with the atmosphere, volcanic actions, and breakdown of metabolites has a prodigious impact on marine flora and fauna (Geist and Hawkins, 2016). Even though the marine biosphere appears to be a nutrient-rich ecosystem however, the actual nutrients available to lead a prosperous life for microbes are limited (Satpute et al., 2010; Merchant and Helmann, 2012). Under such nutrient-deficient and challenging circumstances, marine microbes withstand and compete through the production of a unique magical molecules (Gudiña et al., 2015). Therefore, the marine ecosystem grips an immense assure to invent an innovative bioactive compounds like enzymes, vitamins, antibiotics, drugs, biosurfactant (BS), and bioemulsifier (BE) (Satpute et al., 2010; Gudiña et al., 2016; Waghmode et al., 2019). Marine microbes produce not only diverse types of BSs but also other bioactive molecules under extreme surroundings of salinity, fluctuations in temperatures and pH, availability of limited nutrients, and increased UV exposure (de Carvalho, 2018; Naughton et al., 2019). Studies of marine microbes require access to the sea using well-equipped research ships which is costly and is a major hindrance (Baharum et al., 2010). Therefore, most of the marine microorganisms documented are from mangroves and coastal regions. Due to financial constraints and the inability to grow in common media, ∼0.01 to 0.1% marine microbes are culturable (Baharum et al., 2010; Antunes et al., 2019). Currently, with the advancement of metagenomics, the approaches have been used to identify new genes involved in the secretion of BS (Araújo et al., 2020). In this review, readers are served with an interesting but brief information on overall diversity of BS produced by marine microbes with a particular emphasis on *Planococcus* sp. Genomic insights of *Planococcus* sp. and pathways involved in BS synthesis are considerably evident from the literature. Sparse literature available on the physico-chemical characterization of *Planococcus* BS is included. We also highlight noteworthy applications of *Planococcus*-derived BS in hydrocarbon-oil biodegradation, and biomedical fields.

## Frequently Reported Marine Microbial Communities for Biosurfactant Production

**FIGURE 1:**
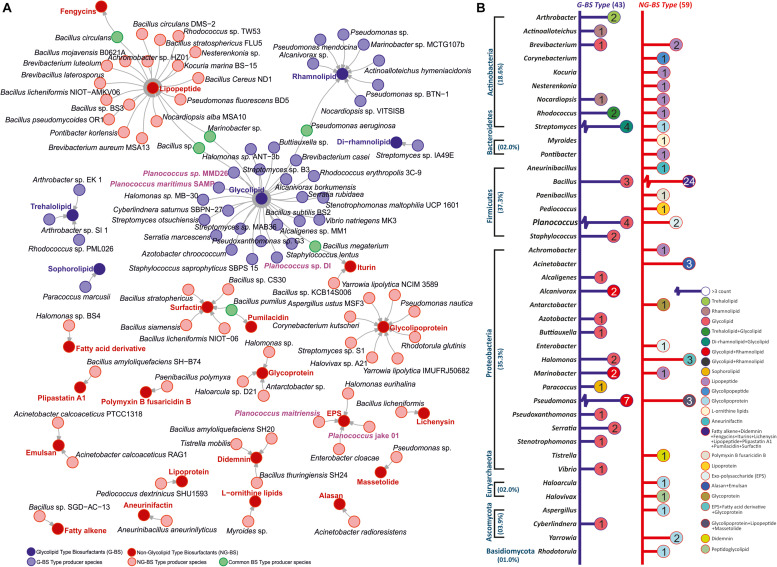
**(A)** Summary of different types of biosurfactant (BS)/bioemulsifier (BE) producing marine micobial species. **(B)** Phylum and genus taxon-based segregation of BS/BE producer literature reports (access till April 2020) from marine origin.

Marine environments have constantly fascinated interest as productive repository port of surface-active biomolecules. This section provides an outline of the respective marine microbial BS producers. Organisms from several phyla like Actinobacteria, Bacteroidetes, Firmicutes, Proteobacteria, Euryarchaeota, Ascomycota, and Basidiomycota have been reported for the prduction of diverse types of BS (Figure 1A). Total ∼102 reports available on BS producing marine bacteria, representing 43 on glycolipid and 59 on non-glycolipid type. Among the six reports available on *Planococcus* sp., four are on glycolipid-type BS and two are on exopolysaccharides (EPS). The majority of the reports on BS are focused on *Bacillus* (25%) and *Pseudomonas* (11%) followed by *Planococcus* (6%) and then *Halomonas* (5%). *Serratia, Arthrobacter, Staphylococcus, Brevibacterium, Acinetobacter*, and *Streptomyces* were reported around 2–3% for novel BS production (Mapelli et al., 2017). Some of the recently reported stains include *Marinobacter* (Tripathi et al., 2019), *Cyberlindnera* (Balan et al., 2019), *Acromobacter* (Deng et al., 2016), *Buttiauxella* (Marzban et al., 2016), and *Paracoccus* (Antoniou et al., 2015). Nevertheless, BS production by marine microbes remains largely unexplored due to the difficulties to cultivate them in the laboratories (Gudiña et al., 2016; Tripathi et al., 2018; McCarthy et al., 2019). The global demand for BS is the foremost driving force to explore untapped marine microbial diversity (Floris et al., 2018; Twigg et al., 2019). There are many other marine microbes where the entire characterization of BS has not been revealed (Figure 1B). The type, quality, and yield of BS are dependent on microorganisms, their growth conditions, carbon, and nitrogen source, stability to withstand fluctuating pH, temperature, salinity, and agitation (Tripathi et al., 2018; Somoza-Coutiño et al., 2020).

## *Planococcus* Species – Untapped Marine Resource for the Production of Biosurfactant and Other Metabolites

*Planococcus* is a Gram-positive, psychrotolerant, halophilic bacterium, which was initially proposed by Migula (1994) in 1894. Despite having such old history, *Planococcus* was not reported in the public domain validly for several years. Kocur et al. (1970) proposed the revised description of the genus *Planococcus*. Initially, there were numerous acceptance and rejections whether to consider these flagellated cocci either under the genus *Planococcus* or *Planosarcina.* From 1949 to 1969, a lot of recommendations were given by several researchers to anticipate flagellated cocci under the roof of genus *Planococcus* considering few features like flagellated structure and % GC content (40–50%) (Kocur et al., 1970). Authors used seven strains having flagellated cocci isolated from a marine source and carried out morphological, cultural, biochemical, and physiological characteristics along with sensitivity to antibiotics and placed them under the genus of *Planococcus* (Van Hao and Komagata, 1985). The small cocci structures which were previously accommodated as a *Micrococci* group are presently popular as *Planococcus* (Cole, 1990; Engelhardt et al., 2001). This is how the successful journey of classification for the genus *Planococcus* began by considering the size (∼1.4 μm) and cocci (short) shape. *Planococcus* grows at 25°C within 3–5 days, results in the formation of orange, round, umbonate colonies (Mykytczuk et al., 2013). In 2001, the novel hydrocarbon-degrading *P*. *alkanoclasticus* sp. nov. strain MAE2 was isolated and characterized by the sediment of the intertidal beach (Engelhardt et al., 2001). This work was dedicated toward the complete characterization of novel strain by growing in a Bushnell-Haas medium enriched with crude oil (0.5 g). This novel strain was compared with three other species of *Planococcus*, namely *Planococcus kocurii* (NCIMB 629^T^), *Planococcus citreus* (NCIMB 1493^T^), and *Planococcus okeanokoites* (NCIMB 561^T^) through fatty acid composition and genotypic study (mol % G + C determination, sequencing of 16S rRNA gene) (Engelhardt et al., 2001).

*Planococcus* sp. strain S5 degrades aromatic hydrocarbons such as salicylate or benzoate through the production of two noticeable enzymes. The authors noted that when strain S5 was grown on salicylate, it produces two types of dioxygenase enzymes (catechol 1,2-dioxygenase and catechol 2,3-dioxygenase) that catalyze the cleavage of oxidative ring catechol to cis and cis-muconic acid (Łabużek et al., 2003). They characterizd those enzymes by growing *Planococcus* in the presence of high concentration of phenol. Muthukamalam et al. (2017) suggested that enhanced quantity of enzymes produced in presence of hydrocarbon and certainly play a crucial role in degradation process. Abilities of BS production along with these enzyme can compliment microorganisms toward hydrocarbon biodegradation potential.

Kumar et al. (2007) isolated *P. maitriensis* Anita I (designated as NCBI Gen-Bank Accession number EF467308) from the coastline sea water of Bhavnagar district, Gujarat, India. This group reported on EPS production from *P. maitriensis* and its emulsifying efficacy. The strain produced EPS consisting of protein (24.44%), carbohydrate (12.06%), uronic acid (11%) along with sulfate (3.03%). This product was purified through centrifugation, precipitation (isopropanol), dialysis, and lyophilisation techniques. The EPS emulsifies xylene powerfully when compared with standard gums. The cell-free culture supernatant also showed a reduction of surface tension (ST) (72 to 46.07 mN/m) and interfacial tension (IFT) against hexane (45.57 ± 0.07 to 20.96 ± 1.56 mN/m) and xylene (33.84 ± 0.19 to 22.95 ± 0.11 mN/m). Thus, for the first time, physical properties of BS from *Planococcus* appeared in the literature.

Having this information at the background, Ebrahimipour et al. (2014) isolated *Planococcus* sp. from an oil-contaminated area (Iran) and further reported large-scale production of extracellular BS and utilization of crude oil / hexadecane. Analysis of some of the physical properties like emulsification activity, its stability at different temperatures, pH, and salt tolerance broadens the application of BS. Emulsifying activity of BS demonstrated 77% of bioemulsification activity against crude oil.

Major focus of Kavitha et al. (2015) appeared on investigating the influence of the phase-separated disintegration process in pre-treatment by using *P. jake*. This experiment was performed in two phases. For the first time authors had demonstrated the effects of CaCl_2_ (0.06 g/g SS) on deflocculation and further pretreatment with BS producing bacteria in phase separation for biodegradation process. Authors had assessed BS production abilities of culture through various screening assays.

In 2016, Ganapathy et al. (2016) isolated *P. maritimus* MKU009 from Pichavaram (South East Coast), India, and identified the methyl glucosyl-3,4-dehydro-apo-8-lycopenoate as a novel carotenoid. Four genes (*crtN, crtB, crtP, crtI*) were reported for carotenoid production located in the first contig of the genome assembly. Such genome-level information is certainly useful to reveal the functional genomics of carotenoid synthesis and metabolic engineering (Han et al., 2019). Thus, metabolic engineering approaches would be supportive to explore additional genetic level information of bioactive metabolites including BS. Yang et al. (2018) had isolated a *Planococcus* culture from oil-contaminated soil, Qinghai-Tibetan Plateau. Genomic research on *Planococcus* strain Y42 illustrated the theoretical mechanism of oil degradation which would be helpful for biodegradation of various hydrocarbons.

Recently, we reported *P. maritimus* strain SAMP for BS production. Based on the physical properties like critical micelle concentration (CMC), ST, IFT, CA (contact angle), wetting properties, and emulsification index proved the BS as a powerful substance. The CMC value of terpene containing BS was found to be 1.3 mg/mL and reduced ST of PBS from 72 to 30 mN/m. The analytical characterization revealed the existence of sugar and lipid moieties in BS. Possibly this was the first report detailing the physico-chemical properties of BS derived form *Planococcus* sp. (Waghmode et al., 2019).

Response surface methodology (RSM), a statistical Technique is being routinely used to design experiments, build models, evaluate the effect of an interactive variables to plot their optimal conditions and enhance the yield of microbial products. In recent times, Hema et al. (2019) used RSM to enhance the yield of BS (glycolipid) from the wild strain, *Planococcus* MMD26.

## Bioprospecting of *Planococcus* and Its Biosurfactants, Exopolysaccharide, Bioactive Compounds for Industrial Applications

Biosurfactants have an extensive scope in diverse industries such as pharmaceutics, cosmetics, food, agriculture, textile, petroleum, and wastewater. BSs are well identified as multi-purposeful agents, as antimicrobial, emulsifying, stabilizing, wetting, and moisturizing. Currently, *Planococcus*-derived BSs attained a prospective significance for environmental applications in remediation of organic/inorganic contaminants, pharmaceutical products, and microbial-enhanced oil recovery (MEOR).

Various microbial communities can efficiently degrade water-insoluble substrates like oils, hydrocarbons through different mechanisms. Firstly, organisms adhere to the oil droplets, aggregating around, and then solubilize them by breaking into smaller chains (Bach and Gutnick, 2004). After creating these miniature droplets, microbial cells interact more efficiently and subsequently degrade hydrocarbons (Karlapudi et al., 2018; Paniagua-Michel and Fathepure, 2018) through BS/BE (Banat et al., 2014). It is an imperative to note that being a marine strain, researchers have mainly targetted the genus *Planococcus* and/or its BS for bioremediation purposes. Engelhardt et al. (2001) highlighted the abundance of *Planococcus* sp. MAE2 in a variety of marine/oceanic surroundings, including few Antarctic niches. Authors reported the potential of MAE2 strain in the biodegradation of branched n-alkanes present in crude oil (C11 to C33), but not aromatic hydrocarbons. This study was not associated with any kind of BS production. Thus, the existence of such hydrocarbon-degrading *Planococcus* sp. is extremely valuable to control contamination by hydrocarbons in marine reservoirs (Park et al., 2018; Somee et al., 2018). Similarly, the strain S5 has been described for degradation of aromatic hydrocarbons like salicylate or benzoate. The S5 strain possesses plasmid (pLS5) having the capacity to degrade salicylate through a metabolic pathway (Łabużek et al., 2003), and the fact was proved through curing experiment. Supportive data for degradation of oil also appeared from the genomic analysis (Yang et al., 2018). Authors have successfully used the *Planococcus* Y42-derived BS for bioremediation and oil recovery purpose. This work illustrated the exploitation of *Planococcus* strains for hydrocarbon degradation through understanding the draft genome information (Engelhardt et al., 2001; Jung et al., 2018; Waghmode et al., 2019). The genome of *Planococcus* sp. PAMC21323 contains the detoxification enzymes of nitroalkanes, aromatic hydrocarbons, and heavy metal ions. *Planococcus* sp. PAMC21323 has three extradioldioxygenases (Plano 0315, 2898, and 2901) that catalyze the cleavage of aromatic ring structure. Therefore, the genome sequence of this psychrotrophic strain showed the potential application for bioremediation in harsh environments (Santos et al., 2016; Jung et al., 2018).

In addition to bioremediation of hydrocarbons, *Planococcus* sp. are also known to participate in MEOR processes. Ebrahimipour et al. (2014) endorsed *Planococcus* sp. for large-scale production of BS and proposed as an extraordinary aspirant for MEOR. Production of BS is associated with the growth of the organism and it has an advantage for a one-stage continuous culture system during scale-up processes.

*Planococcus* sp. has been reported for production of biopolymer, EPS. In 2007, Kumar et al., proposed that the EPS produced by *P. maitriensis* Anita I can spread oil and is equivalent in properties with well-known mild nonionic synthetic detergents like Triton X-100 and Tween 80. Therefore, authors recommended its applications in bioremediation, MEOR and cosmetics.

Researcher have utilized the functionality of *Planococcus* for biogas production. Kavitha et al. (2015) isolated a new strain *P. jake* 01 and demonstrated its application for biogas production. The, pre-treatment procedures in biogas production through bacteria are significant to diminish the suspended solids and chemical oxygen demand of deflocculated pre-treated sludge. These processes are highly efficient than flocculated sludge (treatment with bacteria only).

Biosurfactants are the most significant and precious microbial products. In the medical field, the BSs act as antibacterial, antiviral, antifungal, anticancer, antioxidants, anti-adhesive, immunomodulator, stimulate dermal fibroblasts, vaccines, and gene therapy (Williams, 2009; Manikandan and Senthilkumar, 2017). In 2016, Ganapathy et al. (2016) reported the production of methyl glucosyl-3,4-dehydro-apo-8-lycopenoate as a novel carotenoid with antioxidant property from *P. maritimus* MKU009 strain. The foremost study reported by us is on cytotoxic activity of terpene containing *Planococcus*-derived BS against HeLa, MCF-7, and HCT cells lines at concentrations 41.41 ± 4.214 μg/mL, 42.793 ± 6.072 μg/mL, and 31.233 ± 5.083 μg/mL, respectively. The BS was also exploited against *Plasmodium falciparum* to determine the killing efficacies of the whole organism. Thus, we have reported the EC_50_ (34 μM ± 0.26) value against *P. falciparum*. BS also exhibited anti-tubercular activity IC_50_ value at 64.11 ± 1.64 μg/mL and MIC at 160.8 ± 1.64 μg/mL concentration (Waghmode et al., 2020). Exploring such applications of *Planococcus*-derived BS is noteworthy and can encourage the researcher to explore BS further for pharmacological applications.

## Functional Genomics Insights of *Planococcus* Species and Biosynthetic Pathway for the Production of Biosurfactant

**FIGURE 2:**
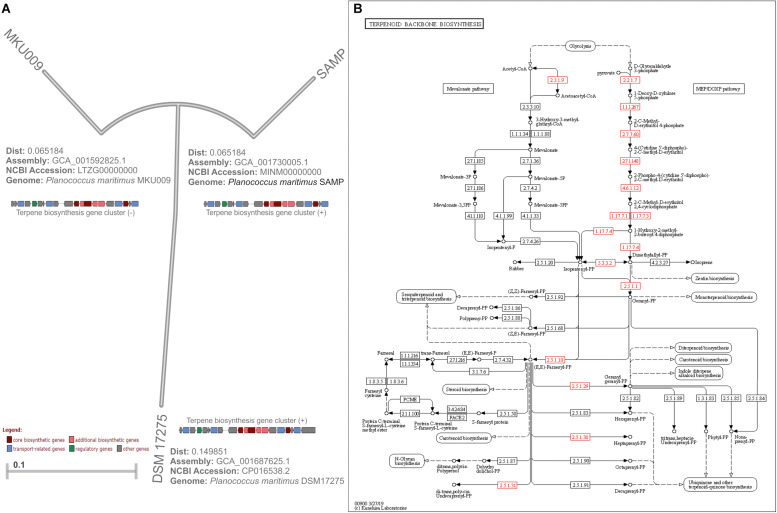
**(A)** Bonafide genome tree of *Planococcus maritimus* genomes available from NCBI database (access till April 2020). **(B)** KEGG database-based metabolic pathway of terpene biosynthesis derived from *Planococcus maritimus* strain SAMP. Note: Part of the gene cluster image adopted from Waghmode et al. (2019).

The genomic study provides profound insight into secondary metabolites produced by various bacterial communities, and functional genomics offers the biochemical characteristics of the bioactive compounds (Van Lanen and Shen, 2006; Shaligram et al., 2016; Waghmode et al., 2017). The whole-genome sequencing study of *P. maritimus* strains revealed an uniform genome size of about 3,220,000 bp for SAMP, 3,280,721 bp for DSM 17275, and 3,251,644 bp for MKU009 (Figure 2A). The similar GC content is reported for all the strains that are about 47.27%. The unique vital genes associated with the SAMP strain was glucose-1-phosphate thymidylyl transferase; for strain DSM 17275, it was cell wall binding repeat two family proteins, and for strain MKU009, it was DUF559 domain-containing protein. The entire gene cluster was screened to identify the secondary metabolite genes. However, the entire gene sets involved in the synthesis of terpene was identified in lipid synthesis pathway. The comparative genome analysis has helped us to identify the genomics potential of all the three strains. The presence of terpene synthesis cluster was observed in genomes of all three strains. From genome analysis, terpene biosynthetic pathway was traced out and it could be the backbone molecule for production of BS (Figure 2B). The location of the gene cluster in all three genomes is different; the entire gene cluster was on the positive strand for two strains DSM17275 and SAMP while for strain MKU009 on the negative strand (Waghmode et al., 2019).

## Conclusion

Marine microorganisms emerged as a rich source for various natural bioactive compounds. Among the marine microbial world, *Planococcus-*derived BS offers great interest to the scientific community due to its outstanding functional properties including ST, IFT, wetting, emulsification, stability to a wide range of pH, temperature, salt tolerance, etc. The pharmacological activities presented by BS suggest its potential usage as multipurpose ingredients in various microbial-based commercial products. Furthermore, sampling and consequent enrichment culturing techniques of BS producing *Planococcus* are essential to explore the potential of BS in many other fields. The accessibility of efficient fast sequencing methods and bioinformatic tools could be helpful in the analysis of biosynthetic pathways. Rapid tracing of the genes involved in BS production and inducers triggering the expression of those particular genes are mandatory to make feasible commercial-scale production. Such metabolic set-up will enable the exposition of the unknown BS biosynthesis pathways of marine microbes. It also facilitates the expansion of synthetic biology-derived concepts to develop competent recombinant strains. These interdisciplinary research tools will contribute toward the identification, production, purification, and application of BS not only from *Planococcus* sp. but also from other microbes for industrial applications.

## Future Prospects

Little is known about the potential of BS production and hydrocarbon degradation by *Planococcus* sp. of marine origin. Few reports discussed the partial physico-chemical characterization of *Planococcus* BS. More efforts are mandatory to evaluate BS production on a larger scale and to find their role in various technologies. More information is essential for (1) predicting the structural details of *Planococcus-*derived BS, (2) identifying their properties, (3) scale-up and (4) cost-effective production studies. To reduce the cost of BS production, commercially feasible biological and engineering solutions can be employed. The use of low-cost substrates appears to be a promising approach to enhance the productivity of BS commercially. More efforts are indispensable to explore marine BS for additional pharmacological applications. Molecular docking studies also provide new avenues to discover the possible target sites for effective interactions of BS. A detailed investigation in this particular area would offer lead molecules for bio-therapeutics. However, applications of marine microbial originated BS in various industrial sectors are awaited and thus provide enormous opportunities to utilize them in food, cosmetics, agriculture, etc.

## Author Contributions

SW, MS, DS, and SS contributed to the literature search, the design of the figure, and the writing of the manuscript. All authors contributed to the article and approved the submitted version.

## Conflict of Interest

The authors declare that the research was conducted in the absence of any commercial or financial relationships that could be construed as a potential conflict of interest.

## Abbreviations

BS: biosurfactant
BE: bioemulsifier
CMC: critical micelle concentration
CA: contact angle
EPS: exopolysaccharide
IFT: interfacial tension
MEOR: microbial-enhanced oil recovery
RSM: response surface methodology
ST: surface tension.
